# Understanding the Activation of Platelets in Diabetes and Its Modulation by Allyl Methyl Sulfide, an Active Metabolite of Garlic

**DOI:** 10.1155/2021/6404438

**Published:** 2021-10-19

**Authors:** Navya Malladi, Ebin Johny, Shravan K. Uppulapu, Vikas Tiwari, Md Jahangir Alam, Ramu Adela, Sanjay K. Banerjee

**Affiliations:** ^1^Department of Biotechnology, National Institute of Pharmaceutical Education and Research, Guwahati, 781101 Assam, India; ^2^Department of Pharmacy Practice, National Institute of Pharmaceutical Education and Research, Guwahati, 781101 Assam, India

## Abstract

**Background:**

Diabetes mellitus (DM) is a chronic metabolic disorder associated with higher risk of having cardiovascular disease. Platelets play a promising role in the pathogenesis of cardiovascular complications in diabetes. Since last several decades, garlic and its bioactive components are extensively studied in diabetes and its complications. Our aim was to explore the antiplatelet property of allyl methyl sulfide (AMS) focusing on ameliorating platelet activation in diabetes.

**Method:**

We used streptozotocin- (STZ-) induced diabetic rats as model for type 1 diabetes. We have evaluated the effect of allyl methyl sulfide on platelet activation by administrating AMS to diabetic rats for 10 weeks. Flow cytometry-based analysis was used to evaluate the platelet activation, platelet aggregation, platelet macrophage interaction, and endogenous ROS generation in the platelets obtained from control, diabetes, and AMS- and aspirin-treated diabetic rats.

**Results:**

AMS treatment for 10 weeks effectively reduced the blood glucose levels in diabetic rats. Three weeks of AMS (50 mg/kg/day) treatment did not reduce the activation of platelets but a significant (*p* < 0.05) decrease was observed after 10 weeks of treatment. Oral administration of AMS significantly (*p* < 0.05) reduced the baseline and also reduced ADP-induced aggregation of platelets after 3 and 10 weeks of treatment. Furthermore, 10 weeks of AMS treatment in diabetic rats attenuated the endogenous ROS content (*p* < 0.05) of platelets and platelet macrophage interactions. The inhibition of platelet activation in diabetic rats after AMS treatment was comparable with aspirin treatment (30 mg/kg/day).

**Conclusion:**

We observed an inhibitory effect of allyl methyl sulfide on platelet aggregation, platelet activation, platelet macrophage interaction, and increased ROS levels in type 1 diabetes. Our data suggests that AMS can be useful to control cardiovascular complication in diabetes via inhibition of platelet activation.

## 1. Introduction

Diabetes mellitus (DM) is one of the chronic and complex disorders among all metabolic diseases. “Diabetes mellitus” is characterized by persistent hyperglycemia with disturbances of carbohydrate, fat, and protein metabolism resulting from defects in insulin secretion, insulin action, or both. Changes in lifestyle and rapid urbanization have increased the incidence of diabetes and its prevalence day by day. According to the International Diabetes Federation (IDF), 463 million adults (20–79-year age) are diabetic in the year 2019 with a record of 4.2 million deaths. Further, IDF interprets that there could be still more diabetic patients by the years 2030 and 2045 with an estimation of 578.4 and 700.2 million cases, respectively [1].

Type 1 diabetes is a condition that resulted from insufficient production of insulin by the pancreas while type 2 is due to improper utilization of produced insulin and ultimately leads to higher glucose levels in the circulatory system [2]. Both type 1 and type 2 diabetes require careful monitoring and control of blood glucose levels, if not this uncontrolled condition over time may root to several complications including cardiovascular disease (CVD). Cardiovascular complications in diabetes occurring as the disease progresses and leads to premature mortality [3–5]. IDF has suggested that diabetic patients are having 2-3 times more probability of getting CVDs than nondiabetic patients [6]. The central mechanism for these cardiovascular complications in diabetes includes an imbalance in the systems which maintain the homeostasis of blood coagulation and fibrinolysis [7]. This imbalance results in diabetic thrombocytopathy [8], a condition that majorly affects platelet function and ultimately results in heart attacks or stroke in diabetes [9–12]. Scientific research has shown that antiplatelet therapy can reduce cardiovascular complications in diabetes and premature death [13, 14].

Despite having many drugs and therapies for the management of diabetes, it is still under the category of life-threatening diseases because of its complications. Reducing these cardiovascular complications in diabetes has become more challenging than controlling the disease itself. This has made an immense impact on scientific researchers for exploring new strategies for diabetic treatment. Dietary therapy in diabetes is one among them showing a tremendous effect on preventing as well as controlling diabetes [15, 16].

Among large numbers of nutritional diets, garlic has been showing a very promising effect on diabetic as well as diabetic complications [17–21]. An antiplatelet property has been observed in raw garlic, preventing cardiovascular complications in diabetes [22–24]. Scientific literature strongly supports that garlic is showing these beneficial effects mainly due to its sulfur-containing compounds [25–27]. Allyl methyl sulfide (AMS), an active metabolite observed inside the body after oral administration of raw garlic, has been shown to effect diabetes by reducing glucose levels, increasing insulin levels, and reducing hepatic oxidative stress caused by glucotoxicity in diabetes [28, 29]. However, the effect of AMS on diabetic complications in thrombosis especially on platelet activation and aggregation is yet to be reported.

Therefore, in this present study, we aim to understand the various altered parameters of platelets isolated from diabetic rats and the effect of garlic metabolite, allyl methyl sulfide, on altered parameters of diabetic platelet.

## 2. Materials and Methods

### 2.1. Reagents

Allyl methyl sulfide (cat no. A34201-25G) and phorbol 12-myristate 13-acetate (PMA) (cat no. P8139-1MG) were obtained from Sigma (St. Louis, Missouri, USA). Flow cytometry antibodies such as CD61 FITC antibody (cat no. 104305) and APC anti-mouse/rat CD62P (P-selectin) antibody (cat no. 148303) are from Bio Legend (San Diego, California, USA), and CD14 PE antibody (cat.no: 561707) is from BD Bioscience (Franklin Lakes, NJ, USA). Adenosine diphosphate (ADP) was obtained from Hi-Media (cat no. RM437-1G). H_2_DCFDA dye (cat no. C6827) was from Invitrogen. Cell culture reagents such as RPMI-1640 (cat no. 31800-014), antibiotic-antimycotic (100X) (cat no. 15240062), and fetal bovine serum (cat no.10270106) are from Gibco.

### 2.2. Animals and Study Design

Wistar rats 200-250 g were used to develop diabetes and evaluate the effect of AMS on altered platelet function in diabetes. Animals were procured from Jeeva Life Sciences (Hyderabad, India). The study was approved by the Institutional Animal Ethics Committee (IAEC) of National Institute of Pharmaceutical Education and Research (NIPER), Guwahati, India (NIPER/BT/2020/37). The animals were housed in individually ventilated cages (IVC) at an animal house facility of NIPER, Guwahati, under standard conditions (temperature 23 ± 1°C, 50 ± 15% relative humidity, and 12 h light/dark cycle). Wistar rats were allowed free access to food and water ad libitum during the study. Post seven days of acclimatization, animals were randomly allocated into four groups (*n* = 6): group 1: control; group 2: diabetes (STZ 35 mg/kg); group 3: diabetes+AMS (50 mg/kg); and group 4: diabetes+aspirin (30 mg/kg). All these rats were maintained for 10 weeks. Every week, the body weight of all animals was recorded to understand the body weight gain or loss during the experimental period and glucose levels were monitored by using a glucometer (Accu Chek Active, Roche). The doses of AMS and aspirin were chosen from our previous work and scientific literature [30, 31].

### 2.3. Induction of Diabetes

Diabetes was chemically induced in Wistar rats (weight 200-250 g) with streptozotocin. After 6 hours of fasting, adult Wistar rats were administered with a single intraperitoneal (I.P) injection of a freshly prepared solution of streptozotocin (STZ) in ice-cold citrate buffer (0.01 M, pH 4.5) at a dose of 35 mg/kg body weight. Animals were then monitored for the next seven days for their blood glucose levels by using a glucometer (Accu Chek Active, Roche). The induction of diabetes was confirmed by monitoring fasting blood glucose. The rats with >250 mg/dl of blood glucose levels were considered diabetic.

### 2.4. Oral Dosing

Allyl methyl sulfide was administered orally at a dose of 50 mg/kg in corn seed oil for 10 weeks in diabetes Wister rats and referred to as the ‘diabetes+AMS' group. Aspirin was given at a dose of 30 mg/kg in 0.5% carboxy methyl cellulose (CMC) orally to another group of diabetes Wistar rats and referred to as the ‘diabetes+aspirin' group. The control group was referred to the rats feed with corn seed oil.

### 2.5. Cell Culture

Human macrophages (THP-1 cells; an acute monocytic leukemia cell line) were a kind gift from Translational Health Science and Technology Institute (THSTI, Faridabad, India). The cells were cultured in the RPMI 1640 medium enriched with fetal bovine serum (10% *v*/*v*) and antimicrobial agents (antibiotic and antimycotic) (100 U/ml) and cultured under standard conditions that are 37°C and 5% CO_2_ incubator (eppendorf CellXpert C170).

### 2.6. Blood Collection and Isolation of Platelets

The platelets were isolated from blood by dual centrifugation. It has been carried out at room temperature. Preventive measures have been taken to avoid platelet activation during the process. Initially, the animals were anesthetized with the help of isoflurane. Through retroorbital plexus using capillary tube, blood was collected into a tube containing 3.8% sodium citrate (9 : 1 ratio) and centrifuged at 500 rpm for 15 minutes at 20°C temperature. The centrifugation results in isolation of platelet-rich plasma (PRP) as the upper layer in a tube. Almost 3/4^th^ of this layer was taken into a fresh tube using a wide-bore pipette tip. Further, platelets were pelleted by centrifuging the PRP at 400 g for 10 minutes at 20°C. The platelets pellet was washed, resuspended in HEPES-Tyrode buffer.

### 2.7. Measuring the Number of Activated Platelets by Flow Cytometer

The *in vivo* activated status of isolated platelets was measured by flow cytometry using fluorochrome-tagged antibodies. All these works were carried out within 2 hours of blood collection and took all measures to avoid platelet activation during handling of the sample. 7 *μ*l of PRP was taken in a tube and diluted by adding 50 *μ*l of HEPES-Tyrode's buffer having 1% BSA. Samples were added with antibody mix having FITC anti-mouse/rat CD61 antibody (platelet surface marker) and APC anti-mouse/rat CD62P antibody (platelet activation marker) diluted with HEPES-Tyrode's buffer (with 1% BSA). Stained platelets were incubated in dark for 30 minutes at room temperature. Samples were immediately analyzed by using Attune™ NxT Flow Cytometer, where analysis was done by taking 50,000 events for each sample. The compensation was done using individual antibody-stained cells and unstained cells in order to avoid spillover from one channel to other. Platelets were gated on forward light scatter (FSC) vs. side light scatter (SSC) plot, and the percentage of CD62P positive cells is enumerated among platelets positive for CD61 cells which give % activated platelets.

### 2.8. Measuring Platelet Aggregation in Presence of ADP, a Platelet Agonist, by Flow cytometry

With a few modifications to previously mentioned procedure, the hypersensitivity of platelets in presence of platelet-aggregating agents (ADP) has been observed by measuring the percentage aggregation of platelets by flow cytometry [32]. 50 *μ*l of freshly isolated platelet-rich plasma (PRP) was resuspended in 450 *μ*l of HEPES-Tyrode buffer. Platelets were activated by adding 20 *μ*M ADP as a final concentration and incubated for 10 minutes at 37°C under shaking conditions at 1000 rpm using a thermoshaker. Diluted PRP without agonist was used as a negative control. Cells were analyzed by flow cytometry for the presence of platelet aggregation. Platelets were differentiated from platelet aggregated by gating cells on forward light scatter (FSC) versus side light scatter (SSC) plot where the platelet aggregates are having increased size and density.

### 2.9. Analysis of Platelet and Macrophage Interaction by flow cytometry

The heterogeneous interactions of platelets with macrophage were analyzed by flow cytometry method using phorbol 12-myristate 13-acetate- (PMA-) differentiated THP-1 macrophages [33, 34].

For monocyte differentiation to macrophage, THP-1 monocytes (1.5 × 10^6^ cells) were seeded in 6-well plate having 1 ml of Roswell Park Memorial Institute medium (RPMI-1640) which was supplemented with 100 *μ*g/ml streptomycin, 100 U/ml penicillin, and 10% fetal bovine serum. The monocytes were differentiated to macrophages by adding 50 ng phorbol 12-myristate 13-acetate (PMA) and incubated for 48 hours in an incubator maintaining 5% CO_2_ at 37°C under humidified conditions. After 48 hours of incubation, media was changed to fresh RPMI-1640 complete media without the addition of PMA and incubated for 48 hours in an incubator.

For macrophage and platelet coculture study, isolated PRP of 7 *μ*l was added in a culture plate having macrophages derived from THP1 and incubated for 30 minutes in a CO_2_ incubator. After successive coculturing of platelets, each well has been washed with PBS to remove floating platelets. Cells were trypsinized by adding 0.25% trypsin EDTA and incubated in a CO_2_ incubator for 2 minutes. The detached cells were collected in an individual tube and centrifuged at 400 g for 10 minutes at 20°C. The supernatant was discarded; the pellet was washed with PBS and fixed by incubating with 4% paraformaldehyde for 10 minutes. The cells further were washed and resuspended in PBS for labelling with fluorescently conjugated antibodies of macrophage surface marker (CD14 PE) and platelet-specific marker (CD61 FITC). The cells were incubated in dark for 30 minutes at room temperature and analyzed by Attune™ NxT Flow Cytometer. Analysis was done by gating the macrophage population under FSC vs. SSC plot. To compensate for the overlapping spectra single-stained cells were used. Finally, we measured the mean fluorescence intensity of CD61-FITC in macrophage (CD14+) population inorder to quantify platelet macrophage interaction.

### 2.10. Measurement of Endogenous ROS in Platelets

Intracellular ROS was measured by using 2′,7′-dihydrodichlorofluorescein diacetate, H_2_DCFDA, dye as per the procedures mentioned in articles [35, 36]. The freshly isolated PRP (7.0 *μ*l) was diluted in 100 *μ*l of HEPES-Tyrode's buffer and incubated with 10 *μ*M of H_2_DCFDA dye under dark conditions for 30 min at 37°C. Later, samples were further diluted with 200 *μ*l HEPES-Tyrode's buffer and mixed gently with a pipette. The tubes were centrifuged at 400 g for 10 minutes at 20°C for pelleting the cells and discarding the supernatant. Further, the pellet was resuspended by adding 100 *μ*l of HEPES-Tyrode's buffer. The unstained samples were used as a negative control. All of these samples were analyzed using Attune™ NxT Flow Cytometer by taking 10,000 events where the mean fluorescent intensity of DCFDA in platelet population was determined for each sample.

### 2.11. Statistical Analysis

All the statistical analysis was performed using GraphPad Prism version 8.0.2(263) (GraphPad Software, San Diego, CA, USA), and its comparisons were made utilizing ANOVA test followed by Tukey's test post hoc analysis. Results were expressed as mean ± standard error, and a *p* value < 0.05 was considered significant.

## 3. Results

### 3.1. Body Weight and Blood Glucose Level Changes in Rats

**Figure 1:**
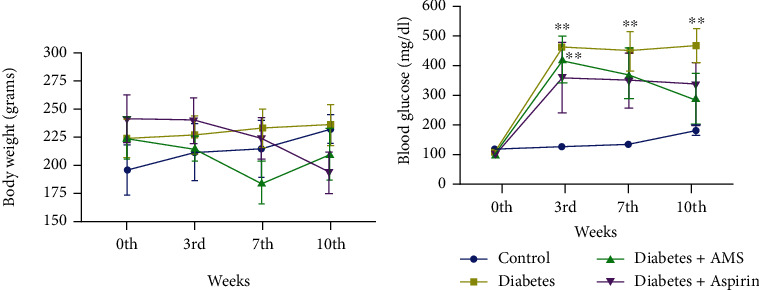
(a) Body weights and (b) blood glucose levels of the control, diabetes, AMS-treated, and aspirin-treated rats at the end of the 0^th^, 3^rd^, 7^th^, and 10^th^ weeks. All values are represented as mean ± SEM (*N* = 4-6). ^∗∗^*p* < 0.01 vs. the control.

We monitored body weights and blood glucose levels of experimental rats until 10 weeks. The mean values of body weight in grams of all study groups were compared in 0^th^, 3^rd^, 7^th^, and 10^th^ week and plotted as a graph. As shown in Figure 1(a), we did not observe any significant body weight changes among all four groups. The blood glucose levels of the diabetes group significantly increased at all (3^rd^, 7^th^, 10^th^ weeks) the time points when compared to the control. However, AMS treatment decreased the glucose levels at 7 and 10 weeks when compared to the 0^th^ and 3^rd^ week of treatment (Figure 1(b)).

### 3.2. Effect of AMS on Platelet Activation in Diabetes

**Figure 2:**
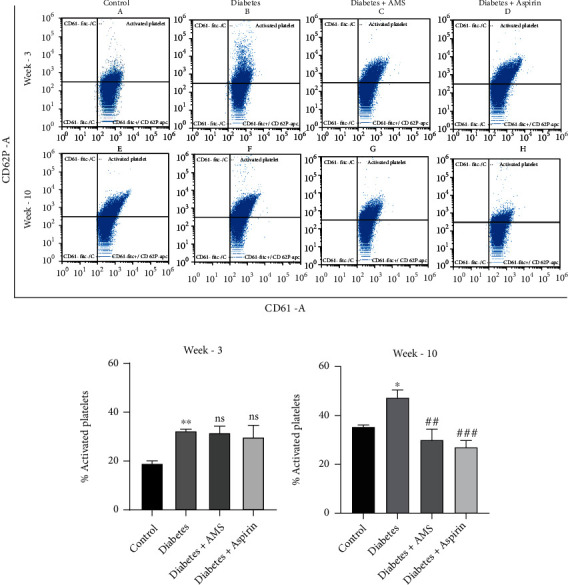
Flow cytometry analysis of platelet activation showing percentage dual positive cells (CD61+, CD62P+). (a) Representation of the scattered plots of percentage platelet activation in the control (A, E), diabetes (B, F), AMS-treated diabetic (C, G), and aspirin-treated diabetic (D, H) groups after 3 and 10 weeks of study. Bar graph showing percentage platelet activation at the end of the 3rd week (b) and 10th week (c). All values are represented as mean ± SEM (*N* = 4-5). ^∗^*p* < 0.05 vs. the control; ^∗∗^*p* < 0.01 vs. the control; ^**##**^*p* < 0.01 vs. diabetes; and ^**###**^*p* < 0.001 vs diabetes; ns: nonsignificant.

The *in vivo* activated status of platelets in the control, diabetes, and treatment groups after 3^rd^ and 10^th^ weeks of study duration has been analyzed by flow cytometry. The graph represents percent CD61 positive on the *X*-axis and CD62P-positive platelets on the *Y*-axis (Figure 2(a)). The dual positive cells are considered activated platelets, and percent of the activated platelets were represented as bar graphs. At the end of 3^rd^ week, the percentage of activated platelets was significantly increased in the diabetes group (~32.4%) when compared to the control (~19.11%). We did not observe any significant changes in the AMS and aspirin-treated groups (Figure 2(b)). Similarly, at the end of 10^th^ week, the percentage of activated platelets was increased significantly in the diabetes group (~47.19%) when compared to the control group (~35.24%). Moreover, both aspirin and AMS treatments significantly decreased this activation of platelets (~30.15 and 25.22%, respectively) induced in diabetes (Figure 2(c)).

### 3.3. Effect of AMS on ADP-Induced Platelet Aggregation

**Figure 3:**
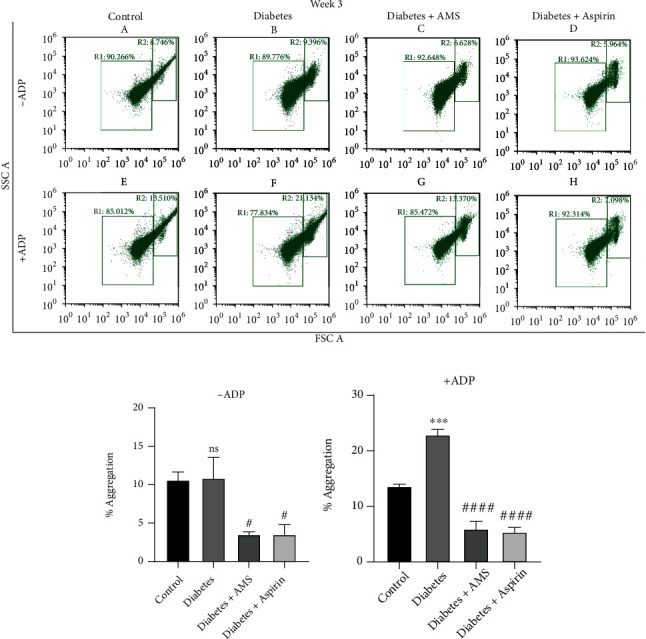
Flow cytometry analysis of platelet aggregation after 3-week treatment. (a) Scatter plot of forward vs. side scatter of platelets from the control (A, E), diabetic (B, F), AMS-treated diabetic (C, G), and aspirin-treated diabetic (D, H) groups, representing percentage of platelet aggregation in the absence and presence of ADP. Bar graph represents percentage of platelet aggregation observed in absence (b) and presence (c) of ADP. All values are represented as mean ± SEM (*N* = 3). ^∗∗∗^*p* < 0.001 vs. the control; ^**#**^*p* < 0.05, ^**####**^*p* < 0.0001 vs. diabetes; ns: nonsignificant.

To assess the effect of AMS on platelet aggregation, flow cytometry analysis was performed on platelets derived from all the experiment groups in the absence or presence of ADP at the 3^rd^ week and 10^th^ week.  Figure 3(a) represents FSC vs. SSC density plots showing the 3^rd^ week platelet aggregation with and without ADP where aggregates are gated as R2. In the absence of ADP, we did not observe any significant change in the percentage of platelet aggregation in the diabetes group compared to the control. However, a significant decrease (3-fold) in percent aggregation was observed in the AMS- and aspirin-treated diabetes groups compared to diabetes (Figures 3(a) and 3(b)). Similarly, as shown in Figures 3(a) and 3(c), we observed a significant induction (~1.6-fold) in platelet aggregation by ADP in the diabetes group when compared to the control. With AMS and aspirin treatment, we observed a significant decrease (~3.8- and 4.2-fold, respectively) in platelet aggregation when compared to the diabetes group.

**Figure 4:**
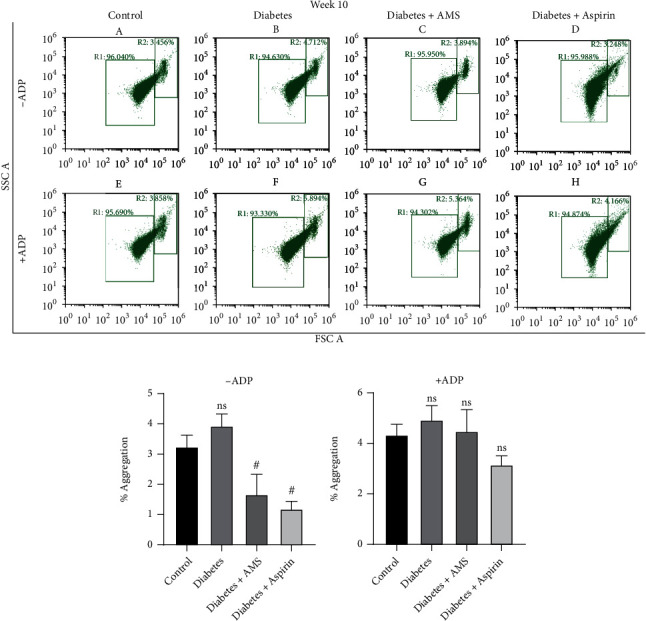
Flow cytometry analysis of platelet aggregation after 10-week treatment. (a) Scatter plot of forward vs. side scatter of platelets from the control (A, E), diabetic (B, F), AMS-treated diabetic (C, G), and aspirin-treated diabetic (D, H) groups, representing percentage of platelet aggregation in the absence and presence of ADP. Bar graph represents percentage of platelet aggregation observed in absence (b) and presence (c) of ADP. All values are represented as mean ± SEM (*N* = 3). ^**#**^*p* < 0.05 vs. diabetes; ns: nonsignificant.

Similar flow cytometry analysis in the presence and absence of ADP was performed at the end of the 10^th^ week, and results were represented as FSC vs. SSC density plots (Figure 4(a)). In the absence of ADP, we observed that the percentage of platelet aggregation increased 1.2-fold in diabetic rats when compared to the control (Figures 4(a) and 4(b)). A significant (*p* < 0.05) decrease in the percentage of platelet aggregation was observed after AMS and aspirin treatment (~2.3- and 3.3-fold, respectively) when compared to diabetes. Similarly, ADP-induced aggregation was increased by 1.1-fold in diabetes while it was decreased by 1.1- and 1.5-fold in the AMS and aspirin treatment groups, respectively (Figures 4(a) and 4(c)).

### 3.4. Effect of AMS on Diabetes-Induced Reactive Oxygen Species Generation in Platelets

**Figure 5:**
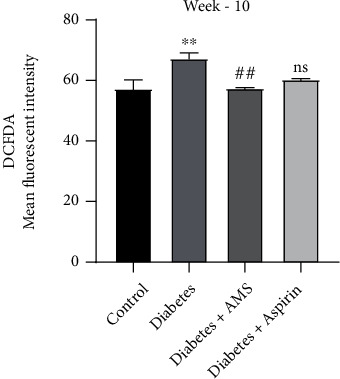
Comparison of ROS levels among the control, diabetes, and treatment groups. All values are represented as mean ± SEM (*N* = 3-4). ^∗∗^*p* < 0.01 vs. the control; ^**##**^*p* < 0.01 vs. diabetes; ns: nonsignificant.

After platelet activation and aggregation study, we measured the reactive oxygen species (ROS) levels in the platelets isolated from the control, diabetic, and treated diabetic rats at 10^th^ week of the study duration. ROS levels in platelets were measured by using 2′,7′-dihydrodichlorofluorescein diacetate (H_2_DCFDA) dye and analyzed it by flow cytometry. H_2_DCFDA dye is a direct measure of amount of ROS present in cells, and the results are expressed as the mean fluorescent intensity (MFI) of DCFDA (Figure 5). Platelet ROS levels was significantly (*p* < 0.05) increased in the diabetic group when compared to the control. However, the ROS levels was decreased significantly (*p* < 0.05) in platelets isolated from the AMS-treated diabetic rats when compared to diabetes rats. We did not observe any significant change in ROS levels in platelets isolated from the aspirin-treated diabetic rats.

### 3.5. Effect of AMS on Diabetes-Induced Macrophage and Platelet Interaction

**Figure 6:**
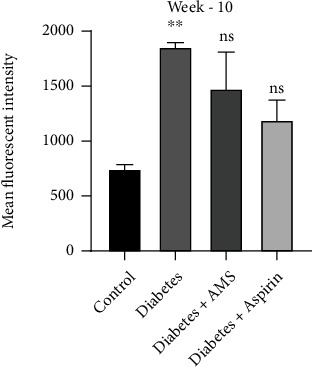
Macrophage platelet interaction in the control, diabetes, and treatment groups. All values are represented as mean ± SEM (*N* = 3-5). ^∗∗^*p* < 0.01 vs. the control; ns: nonsignificant.

Platelet macrophage interaction has been considered an important phenomenon for platelet activation in diabetes [37, 38]. To look at this interaction, macrophage and platelet were incubated together and FACS analysis was performed. THP 1, a well-known monocyte cell, were differentiated with phorbol 12-myristate 13-acetate (PMA) and later cocultured with platelets collected from the control, diabetes, and AMS-/aspirin-treated rats at 10^th^ week of study duration. The results are shown in mean fluorescent intensity of CD61-FITC (marker of platelets) among CD14-positive THP1 macrophages. The data was represented as a bar graph (Figure 6). The increased mean fluorescent intensity was considered an aggregate cell of macrophages and platelets. In our data, the significant (*p* < 0.05) increase (~2.4-fold) in mean florescent intensity of CD61-FITC was observed in macrophages cocultured with diabetes platelets when compared to the control. Although we observed a decrease in macrophage platelet interactions in the AMS and aspirin treatment groups (~1.2- and 1.5-fold) when compared to the diabetic group, these changes were not significant.

## 4. Discussion

Diabetes mellitus is not a single clinical entity but a spectrum of diseases with various diabetic complications [39–41]. Several reports have shown a strong relation between diabetes mellitus and premature cardiovascular events [4, 5]. The underlining mechanisms of cardiovascular complication in diabetes include several physiological and pathological changes in the heart, blood vessels, blood cells, and kidneys [42]. Previous scientific literature identified numerous risk factors i.e., hyperglycemia [43–45], dyslipidemia [46, 47], inflammation [48, 49], endothelial dysfunction, and oxidative stress [50, 51], which together can induce several complexities including cardiovascular complication in diabetes. Studies have also identified that alteration of normal platelet function as one of the major risk factors of diabetic complication and characterized by increased thromboxane synthesis [52], reduced membrane fluidity [53], and increased expression of activation-dependent adhesion molecules (e.g., GpIIb-IIIa and P-selectin) [54]. All of these changes make platelets more reactive and create a prothrombic environment in a diabetes patient [55, 56]. Nevertheless, the studies focusing on platelet dysfunction in type 1 diabetes (T1DM) and its modulation by pharmacological agents are limited. Recent literature says that among all the popular natural remedies, organosulfur compounds from garlic have shown a potential antidiabetic and as well as antithrombotic effect in diabetic individual [17, 18, 22, 23, 57]. Previous research work also supported the role of garlic to attenuate cardiovascular complications in diabetes [18]. Allyl methyl sulfide (AMS) is one of the important sulfur compounds obtained from garlic, and studies showed that AMS is a major metabolite that is detected in the human breath and plasma [28]. Our previous study on AMS suggested that chronic administration of AMS is safe in control rats, where the body weight, food, and water intake along with the histopathology of major organs and serum biomarkers remained normal [30]. Further, the same study showed a beneficial effect of AMS on isoproterenol-induced cardiac fibrosis and dysregulated extracellular matrix (ECM) deposition in the myocardium [30]. Also, our recent finding showed a cardioprotective effect of AMS in pressure overload-induced cardiac hypertrophy and heart failure by ameliorating endogenous antioxidants and mitochondrial function [31]. Further research identified the therapeutic role of AMS on type 1 diabetes where different parameters like blood glucose, HbA1c, oxidative stress, inflammation, and insulinotropic activity were normalised after AMS treatment. All the above parameters remained normal in control rats after AMS administration [29, 58]. However, there is no study to find the effect of AMS on platelet activation. Therefore, in the current study, we determined the major platelet alterations in STZ-induced diabetic rats and explored the antithrombotic effect of AMS, a bioactive derivative of garlic, mainly focusing on platelet activation.

In diabetes, controlling hyperglycemia is the primary goal to reduce complications of diabetes. Poor glycemic control affects platelet activation and vascular dysfunction in diabetes. A previous study reported that a low dose of aspirin reduces blood glucose levels in diabetic rats [59]. In our present study, AMS administration to rats reduced blood glucose levels. The result showed that AMS is more effective to reduce blood glucose level in diabetic rats than aspirin. Our data is supporting the recent studies on AMS, where AMS administered to STZ-induced diabetic rats showed a significant decrease in blood glucose level [58, 60].

Hyperglycemia along with other factors helps to aggregate the platelets in the presence of a small stimulus. Such platelets are referred to as hyperactivated platelets [61]. This hyperactivated platelet has distinct morphology and expresses P-selectin (CD69P) and GP IIb/IIIa receptor on the surface [62–64]. Supporting the previous data, our present study also observed an increase in platelet activation (CD62P levels) in type 1 diabetic rats when compared to nondiabetic rats. This activation of platelets was higher at a later stage (10 weeks) when compared to the initial stage (3 weeks) of diabetes. Moreover, we also observed the effect of AMS on platelet activation. Interestingly, we found a little reduction in platelet activation after initial treatment for 3 weeks with AMS but a significant decrease was found after 10 weeks of treatment. Here, our data suggest that long-term administration of AMS has a superior effect on reduction of platelet activation in diabetes.

Increased platelet activation results in increased platelet aggregation and has been detected in diabetes mellitus [37, 38]. This increased platelet aggregation is a result of increased systemic production of TxA 2 by platelet [65], increased sensitivity of platelets agonist like ADP [66], and impaired production of platelet aggregation inhibitors PGI 2 and NO [67, 68]. The previous finding has shown that the platelet of the diabetes patient was found to be 1.6-fold more sensitive to the ADP-induced aggregation than that of nondiabetes persons [69]. In the present study, we observed the aggregation of platelets (basal level) and its sensitivity to ADP stimuli in both the early (3^rd^ week) and late (10^th^ week) stages of diabetes. At early stages of the disease, there was no difference in the baseline aggregation property of platelets between the diabetes and control groups. However, in the presence of ADP, the diabetic platelets showed an increase in aggregation than the control group. The data suggests that the platelets from diabetic rats were highly sensitive and prone to thrombus formation. A decrease in baseline aggregation was observed in diabetic rats after AMS and aspirin treatment. The percentage of aggregation was also reduced when the platelets were activated by the addition of ADP in treatment groups. The study suggests that AMS has a beneficial effect in reducing the platelet sensitivity and aggregatory properties. Similar to platelet aggregation data in the 3^rd^ week, we observed a decrease in baseline platelet aggregation in the 10^th^ week after AMS and aspirin treatment. Interestingly, in the 10^th^ week of diabetes, we did not observe any significant increase in ADP-induced platelet aggregation when compared to the control. Although we cannot explain the reason for not showing the sensitivity of platelets after ADP addition at week 10, it may be due to desensitization of P2Y1 and P2Y12 ADP receptors of platelets after the long-term interaction with endogenous ADP in chronic diabetes [70]. Further, the control group at 10^th^ week showed higher basal platelet aggregation than the control group at 3^rd^ week. This can be explained by the fact that age itself may enhance the platelet aggregation in the absence of diabetes [71]. After treatment with AMS for 10 weeks, we observed a decrease in ADP induced aggregation. The data overall indicated that inhibition was less at later stages of diabetes and may be due to the alteration of platelet structure and expression of major protein levels that resist the AMS's beneficial effect.

Next, we correlated the platelet aggregation phenotype with intracellular ROS production. Increased ROS has been observed in activated platelets and activates the PKC pathway which led to platelet hyperactivity and aggregation [72]. Supporting the previous literature, our study also found increased ROS content in diabetic platelet when compared to the control. A recent study indicates that AMS administration improved the oxidative stress in STZ-induced hyperglycemic rats [58]. Based on their observation, we evaluated the endogenous ROS generation in platelets after AMS treatment and found an inhibitory effect of AMS on ROS production in platelets.

Increased ROS production can activate the platelets and help to participate in the signaling event of atherosclerosis in diabetes by forming aggregates with monocytes through P-selectin–PSGL-1 interactions [73]. Therefore, platelets play an important role in promoting inflammation in diabetes. The inflammatory condition created by PSGL-1-mediated monocyte activation leads to the synthesis and release of various chemokines, cytokines, and reactive oxygen species. Monocyte platelet interactions also have a role in the coagulation system by a surface expression of phosphatidylserine [74]. Previous literature suggested that increased platelet-monocyte aggregates are the indicator of *in vivo* platelet activation. These aggregates are responsible for the prothrombic stage and play a major role in the development of atherosclerosis in type 1 diabetes [75, 76]. Additionally, macrophage accumulation also plays a vital role in causing diabetic complications [77]. It was reported that a similar interaction between macrophages and platelets causes releases of chemokines and phagocytosis of platelets which further involve in atherothrombosis formation [78, 79].

In the present study, we observed platelet macrophage interactions to evaluate the platelet activation status as well as proinflammatory condition in type 1 diabetes. Here, in the present study, we observed an increase in macrophage and platelet interaction at late stages of diabetes compared to control, whereas after AMS treatment, diabetic rats showed less macrophage and platelet interactions compared to diabetes. The study suggests that AMS can reduce the macrophage and platelet interaction and can further inhibit pro-inflammatory condition which leads to vascular complication in diabetes.

## 5. Conclusion

**Figure 7:**
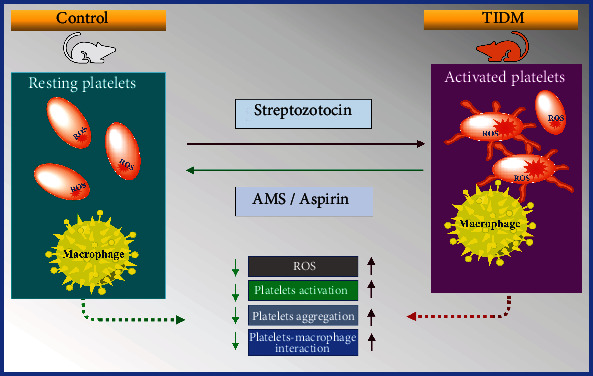
Attenuation of the platelet activation and platelet-macrophage interaction in type 1 diabetes by AMS. AMS: allyl methyl sulfide; ROS: reactive oxygen species; T1DM: type1 diabetes mellitus.

In this study, we found that allyl methyl sulfide inhibits platelet activation and their aggregation in type 1 diabetes. It has also shown an inhibitory effect on increased platelet-macrophage interaction in type 1 diabetes. The increased platelet activation was associated with elevated levels of ROS. Further, our results showed a decrease in ROS levels in platelets after AMS treatment. The summary of the present study has been represented in Figure 7. Overall, this study provides a piece of strong evidence that treatment with AMS could prevent the phenotypic platelet changes seen in type 1 diabetes and may act as a potential therapeutic molecule for cardiovascular complications especially thrombosis in diabetes.

## Data Availability

The data used to support the findings of this study are available from the corresponding author upon request.
